# Management of Cervical Spine Fractures and Injuries: A Literature Review

**DOI:** 10.7759/cureus.75642

**Published:** 2024-12-13

**Authors:** Sanjeevi Bharadwaj, Subasri Balasubramanian

**Affiliations:** 1 Trauma and Orthopaedics, Barts Health NHS Trust, London, GBR; 2 Trauma and Orthopaedics, The Royal London Hospital, London, GBR; 3 Trauma and Orthopaedics, Blizard Institute, Queen Mary University, London, GBR; 4 Emergency Medicine, West Midlands Deanery, Birmingham, GBR

**Keywords:** british orthopaedic association standard for trauma (boast), british orthopaedic association standard for trauma guidelines for spinal clearance, cervical spine fracture, cervical spine injuries, international standards for neurological classification of spinal cord injury (isncsci) impairment scale, national emergency x-radiography utilization study group (nexus) guidelines or the canadian c-spine rule (ccr)

## Abstract

Cervical spine injuries are one of the most common injuries of the spine that are encountered in the emergency department (ED). More than half of all spinal injuries presenting to the ED involve the cervical spine, with nearly half of them resulting from road traffic accidents. The majority of spinal cord injuries are found to occur in males of younger age groups, with almost half of them resulting in incomplete spinal cord injuries. The initial assessment of a patient with a suspected cervical spine fracture would include an Advanced Trauma Life Support (ATLS)-guided documented assessment of the patient involving a detailed, meticulous primary survey followed by a documented assessment of the patient through a secondary survey. Initial management of the cervical spine fracture begins at the scene of injury with prehospital care, which involves immobilisation of the cervical spine with “triple immobilisation” followed by initial airway management in the ED while maintaining in-line neck stabilisation, thereby minimising the neck movements. Neurological assessment according to the impairment scale and imaging in alert and stable patients is done based on established guidelines. However, a CT scan is the gold standard modality of imaging for suspected cervical spine fractures. All patients with a significant mechanism of injury must be assumed to have an unstable injury to their spine until proven otherwise according to British Orthopaedic Association Standard for Trauma (BOAST) guidelines.

## Introduction and background

Cervical spine injuries are one of the most common injuries of the spine, which is encountered in the emergency department (ED) with a nearly 60% incidence among all spinal injuries worldwide, with road traffic accidents accounting for >50% of the mechanism of injury. These injuries encompass a wide spectrum of cervical spine trauma, ranging from those involving cervical soft tissues to those that could result in severe spinal cord injuries such as cervical vertebral fractures and/or intervertebral joint dislocations [1].

A recent systematic review involving 229 studies showed that spinal cord injuries had an incidence of 23.77 per million people worldwide, of which traumatic spinal cord injuries and non-traumatic spinal cord injuries had an incidence of 17.93 and 26.48 per million people, respectively. The study also showed that the incidence was much higher in males with a higher incidence of traumatic spinal cord injury in developing countries than in developed countries [2]. 

The presence of a wide range of motion in the cervical spine, facilitated by a special anatomical osseous-ligamentous combination, makes them more easily prone to injuries [1]. Various mechanisms of injuries have been described based on the forces acting on the cervical spine, such as hyperflexion, hyperextension, axial loading, rotation, and distraction [1]. The commonest mechanism of injury is hyperflexion injury, which involves flexion teardrop fracture, clay-shoveller fracture, facet dislocation (which could be bilateral or unilateral), anterior subluxation, and hyperflexion fracture-dislocation, followed by hyperextension injury, which includes hangman’s fracture, extension teardrop fracture, and axial compression injury, which includes Jefferson’s fracture. The incidence of spinal cord injury is 15-40 per million, with a prevalence of 800 cases/million, with around 80% of them being males and two-thirds of them under the age of 30 years of age. These cervical spine injuries are quite often associated with significant mortality and/or morbidity, although their incidence is found to be rare, with a 30-day inpatient mortality rate ranging as high as 4% to 16.2% and around 21.7% to 32.3% of the patient's deaths within the next one year from the initial inpatient attendance [3-6]. Most cervical spine injuries result in incomplete spinal cord injuries, with an incidence of 42%. However, evidence shows around 29.3% of cervical spine fractures were caused by road traffic accidents, which was also the most common cause in the United States [1, 6-8]. It was also found that 32% of these cervical spine injuries most often occurred at the level of the C2 vertebra, and 20.9% occurred at the level of the C7 vertebra [1, 6-9]. Patient-reported outcomes were suggestive of higher survival rates with nonsurgical conservative management than in those treated with surgical management [3, 6].

## Review

Initial assessment of suspected cervical spine injury

An initial assessment of a patient with a suspected cervical spine fracture would include an Advanced Trauma Life Support (ATLS)-guided documented assessment of the patient involving a detailed, meticulous primary survey followed by a documented assessment of the patient by a secondary survey. According to the British Orthopaedic Association Standards for Trauma (BOAST) guidelines, all patients who undergo spinal clearance following a significant mechanism of an injury must be assumed to have an unstable injury to their spine until or unless proven otherwise [10-15]. The initial assessment of a patient with a suspected cervical spine fracture is aimed at ascertaining the absence of fracture in the cervical spine or identifying an obvious fracture of an occult fracture, which when present, might warrant conservative treatment with a collar or an operative intervention [12]. Tenderness over the spinous processes and across the facet joints of the C1 to T1 vertebrae, discontinuity of the cervical structures on inspection or palpation, and the evidence of haematoma and/or oedema in the vicinity of spinous processes and facet joints should raise suspicion of a cervical spine fracture, although clinical examination has shown to have low sensitivity [12,13]. However, guidelines recommend that all patients should have a documented neurological assessment using an International Standards for Neurological Classification of Spinal Cord Injury (ISNCSCI) impairment scale [12, 14].

American Spinal Injury Association (ASIA) grading, or the modified Frankel scoring system, is a neurological severity scoring system done based on a standardised sensory and motor assessment, which is a universal tool in helping to determine whether the injury is complete or incomplete and also aids in determining the neurological level (motor and sensory) of injury bilaterally [12,16,17]. The examination findings are scored on a scale of A to E, with A reflecting a complete injury of the spine and cord and E reflecting a normal finding [12]. This assessment is performed at regular intervals, from the time of injury to the time of pre-transfer, post transfer, and the immediate postoperative period [14, 17].

Initial management of a patient with a suspected cervical spine injury

The management of the cervical spine fracture begins at the scene of injury with pre-hospital care, which involves immobilisation of the cervical spine with “triple immobilisation”, which has three components, namely, a board, a collar with the head immobilised between two towels or foam wedges reinforced with straps to the forehead and the chin, thus providing the most stable biomechanical immobilisation. This type of immobilisation has also been shown to be the safest way to immobilise while the patients are pending investigations [18, 19]. The first responders need to be well trained and avoid being overenthusiastic to prevent a secondary spinal cord injury, as an excessive movement of the cervical spine is the most common cause of secondary spinal injuries [12, 15].

However, in multiple recent studies, expert consensus argues against spinal immobilisation for fully conscious, neurologically intact patients with penetrating injuries. Even in patients with suspected cervical spine fractures, some experts have argued against the routine use of longboards. However, clinical judgment should be used, and selective immobilisation should be the standard of care [20, 21].

As soon as the patient arrives in the ED, the airway must be ascertained by the anaesthetist, and after the handover is taken from the ambulance crew and paramedics, the patient should be taken off the scoop by bracing from either side one after the other, and the patient should be transferred off the hard spine board to a vacuum mattress to avoid the risk of pressure sores, as they would lead to increased risk of infections in polytrauma patients who may have open injuries in proximity to the sites where pressure sores may occur [22,23]. 

The next important aspect to consider in polytrauma patients with suspected cervical spine fractures is the risk of deterioration while they have been immobilised. The deterioration may occur in respiration or consciousness due to alterations in the levels of inspired oxygen due to airway compromise, where airway support through intubation is needed while maintaining in-line neck stabilisation, thereby minimising neck movements. There is also evidence to support the use of a device called a light wand, which minimises the movements of the cervical spine while intubating [12,24]. 

All patients should have a documented neurological assessment using an ISNCSCI impairment scale [12,14].

Although evidence as per the National Acute Spinal Cord Injury Study (NASCIS) suggests that administration of high-dose methylprednisolone within 3 hrs to 24 hrs improves the patient’s neurological status [25], it is not administered in the United Kingdom as it is believed that administration of steroids has more risks than benefits.

It is important to avoid hypotension and to stabilise the patient’s perfusion well with adequate hydration with intravenous fluids while considering a transfer of a spinal cord-injured patient to the intensive care unit, as they may deteriorate neurologically due to hypoxemia, hypotension, pulmonary dysfunction, and cardiovascular instability [26]. We should aim to maintain a MAP of 85-90 mm Hg in the first week after spinal cord injury.

The decision for imaging in alert and stable patients is taken based on National Emergency X-Radiography Utilization Study Group (NEXUS) guidelines or the Canadian C-Spine Rule (CCR), which suggests that an X-ray of the cervical spine is not unnecessary if there is no tenderness over the spinous process and across the facet joints with normal neurological findings, without painful distracting injuries in a patient who is not intoxicated with no history of altered alertness post injury [12,27,28]. Izzo et al. found that plain anteroposterior, cross-table lateral, and open-mouth odontoid views missed 61% of all fractures and 36% of subluxations and dislocations [12,29]. The same study also showed that plain anteroposterior, cross-table lateral, and open-mouth odontoid views gave false-negative results in 23% of the patients, and half of those patients were found to have unstable cervical spine injuries [12,29]. In patients with low Glasgow Coma Scale (GCS) scores and those with low suspicion of cervical spine fracture, radiographs have been found to have low sensitivity and less usefulness. In a study by Lange et al., CT scans were found to have an overall positivity rate of 6.4% compared to plain radiographs [12, 30]. Computed tomography scans have superseded plain radiographs as the gold standard investigation for any spinal trauma with a suspected cervical spine injury [12,31]. Magnetic resonance imaging (MRI) is used if a CT scan is abnormal, if the patient has abnormal neurology, and for preoperative planning in cases where a patient needs operative management [32-34]. However, MRI is disadvantageous in that it is time-consuming and poses a logistic challenge when scanning patients who need constant monitoring [12,35].

Over the last decades in the recent past, the necessity to improve accuracy and clarity of communication with regard to spinal injuries among clinicians has resulted in the evolution of several clinical classifications of spinal injuries. These classifications were found to be radiographically theoretical and morphological in the description of the injury; therefore, they could not grade the severity of the injury. Hence, they have been unable to predict stability and outcome [12,36]. However, recent classification systems have been found to be more clinically consequential while being more valid and reliable in the guidance of treatment and determination of possible outcomes. Holdsworth, in 1970, came up with a two-column concept classification system that described the injuries based on those involving the anterior column and those involving the posterior column [37]. Allen et al., in 1982, classified based on the mechanism of injury [38]. Harris et al., in 1986, also came up with a similar classification based on the mechanism of injury and injury pattern [39]. However, there is no actual evidence about their reproducibility or validity. However, over the period, the improvement in the imaging modalities has enabled us to assess the injuries based on the pattern of the injuries that we can view on the scans. Over the first decade of the 21^st^ century, Vaccaro et al. (2007) with the Sub-axial Cervical Spine Injury Classification System (SLIC) and Anderson et al. (2007) with the Cervical Spine Injury Severity Score (CSISS) came up with classification systems that are based on modern imaging techniques in combination with neurological injury [40, 41]. The studies on intraobserver and interobserver reproducibility and validity of these two new classifications showed that there were reasonably good intraclass correlation coefficients of 0.71 and 0.883 for Vaccaro et al. and Anderson et al., respectively. Although the CSISS classification system by Anderson et al. is a great tool for research purposes, its complexity makes it difficult for it to be used for day-to-day assessments of patients. This leaves the SLIC by Vaccaro et al. as the best classification system to be used for day-to-day assessment to identify the patients who would need urgent treatment and to determine the threshold for surgical intervention.

Sub-axial Cervical Spine Trauma

The SLIC system is based on the morphology of the fracture, the integrity of the disco-ligamentous complex, and neurological status. Scores of one to three can be treated with non-surgical treatment; a score of four could be treated both surgically or non-surgically, and a score of five and above would need definitive surgical fixation [40]. Although remembering the scores and scoring in the ED might be practically difficult in a busy setting, it is more important to remember the three broad components and the principles that govern the classification system and the decision-making.

Atlantoaxial Cervical Spine Trauma

Atlantoaxial trauma includes injuries to the C1 vertebra, which is the atlas, and the C2 vertebra, which is the axis. In 1919, Geoffrey Jefferson described the fracture of the atlas and classified it into two types based on the integrity of the transverse atlantal ligament (TAL) [42], with type 1 having an intact TAL and type 2 injury in which the ligament was not intact with an atlas-dense interval (ADI) of >3 mm when observed on a lateral view plain X-ray and >7 mm of lateral mass overhang when viewed on an open mouth view X-ray or a coronal view CT of the cervical spine, which would be deemed as an inherently unstable fracture and would warrant an urgent surgical treatment [42].

Two types of fractures can occur at the level of the C2 vertebra, or axis. These include the traumatic spondylolisthesis of the axis, the hangman’s fracture, and the odontoid peg fracture.

The traumatic spondylolisthesis of the axis, or hangman’s fracture, is a fracture occurring bilaterally through the pars interarticularis, i.e., a fracture passing through the ring of the C2 vertebra, which results in the slipping of the C2 anteriorly over the C3, thereby resulting in instability between the C2 and C3 vertebrae. Levine and Edwards, in 1985, described this fracture through a classification system that was based on the angulation of the odontoid peg and the translation of the C2 vertebra over C3 [43]. It has three subtypes, each with an increasing grade of severity. The more angulation of the odontoid peg and the translation of C2 over C3, the higher the grade of the injury, and the higher the grade, the more likely the need for an urgent surgical fixation [44].

The odontoid peg fracture is classified according to the Anderson and D’ Alonzo classification system described by them in 1974 [44]. There are three types, based on the site of the fracture on the dens process of the axis vertebra. Type 1 is the fracture at the tip of the dens and is the rarest of the 3; type 2 is the commonest and is the fracture through the base of the dens process and has high nonunion and complication rates; and type 3, which is the fracture through the body of the axis vertebra, is less common than type 2. Out of these three types, type 2 needs surgical fixation, whereas types 1 and 3 can be managed conservatively [44].

Treatment modalities for the management of cervical spine injuries

Non-operative or Conservative Treatment

Conservative non-surgical modalities played an important role until the advent of recent advancements in surgical management, which limited their use. They are indicated in specific types of cervical spine injuries, such as stable spine fractures and/or in the absence of intervertebral joint dislocations, which could result in instability. However, they have limitations in specific cases, such as delayed recovery or higher recurrence of instability, where they cannot be useful. Patient-specific factors such as age and comorbidities also play an important role in determining suitability for conservative versus surgical treatment. Non-operative conservative options involve the application of the traction principle by employing devices such as external fixators, halo vests and cervical braces [12]. 

Skeletal Skull Traction

This device is especially useful where there is a need for realignment and immobilisation of the high cervical spine following subluxation or dislocation of the intervertebral facet joint and/or burst fractures; however, it is contraindicated in cervical spine injuries with a distraction component [12,45]. Although traction devices are contraindicated, they can be useful in the definitive management of unilateral and/or bilateral facet joint dislocation patients with normal GCS, for whom serial neurological examinations can be repeated [12,45]. This is supported by the recommendation for closed reduction by traction, which suggests that it is prudent to apply initial traction by applying a weight of 10 to 15 pounds, sequentially increasing the weight by five to 10 pounds for each level of injury with serial neurological examinations [12,46]. However, it is important to perform an MRI in patients with low GCS before attempting manipulations or closed reductions of cervical spine injuries, as there is a high chance of progressive worsening of the condition of the injury [12,46].

Operative Management

Operative options include anterior corpectomy and/or discectomy, anterior decompression with fusion, posterior stabilisation, and/or decompression with an aim to provide stability and realignment and also to restore the altered biomechanics of the spine [12,47]. Evidence shows that the results from either of these approaches are equivocal, with no difference in long-term patient-reported outcomes, though severe instability may need a combination of both approaches [48].

A recent systematic review and meta-analysis published in 2023 also showed similar functional outcomes with anterior corpectomy and/or discectomy, anterior decompression with fusion, and posterior stabilisation and/or decompression. However, the study did show that anterior corpectomy and/or discectomy, anterior decompression with fusion, was beneficial as it had less possibility of surgical site infections, C5 palsy, and bleeding while having higher possibilities of dysphagia [49]. 

## Conclusions

Cervical spine injuries are some of the most common spine injuries due to hyperflexion injury. Triple immobilisation is vital in providing the most stable biomechanical immobilisation. It is important to transfer the patient onto a soft foam board as early as possible to avoid pressure sores. The initial assessment of a patient with a suspected cervical spine fracture should include an ATLS-guided documented assessment of the patient involving a detailed, meticulous primary survey followed by a documented assessment of the patient through a secondary survey. Initial management of the cervical spine fracture begins at the scene of injury with pre-hospital care, which involves immobilisation of the cervical spine with triple immobilisation followed by initial airway management in the ED while maintaining in-line neck stabilisation, thereby minimising the neck movements. Computed tomography scan is the gold standard modality of imaging for suspected cervical spine fractures. All patients involved in a major trauma with a significant mechanism of injury must be assumed to have an unstable injury to their spine until or unless proven otherwise according to BOAST guidelines.
